# Association of Life-Course Stressful Life Events with Incident Chronic Kidney Disease: The Potential Mediating Role of Frailty

**DOI:** 10.3390/healthcare14203438

**Published:** 2026-10-14

**Authors:** Chuxuan Luo, Kongwen Lin, Raojuan Chen, Keng Ye, Shanhong Shi, Menglan Huang, Yanfang Xu

**Affiliations:** 1 Department of Nephrology, Blood Purification Research Center, The First Affiliated Hospital, Fujian Medical University, Fuzhou 350005, China; chuxuan_luo@fjmu.edu.cn (C.L.); 2230810681@fjmu.edu.cn (K.L.); raojuan010308@fjmu.edu.cn (R.C.); yekeng2026@fjmu.edu.cn (K.Y.); 2024010032@fjmu.edu.cn (S.S.); huangmenglan2024@fjmu.edu.cn (M.H.); 2 Research Center for Metabolic Chronic Kidney Disease, The First Affiliated Hospital, Fujian Medical University, Fuzhou 350005, China; 3 Department of Nephrology, National Regional Medical Center, Binhai Campus of the First Affiliated Hospital, Fujian Medical University, Fuzhou 350212, China; 4 Central Laboratory, The First Affiliated Hospital, Fujian Medical University, Fuzhou 350005, China

**Keywords:** chronic kidney disease, cohort study, frailty, mediation analysis, stressful life events

## Abstract

**Background/Objectives**: Our research sought to explore the association between life-course stressful life events (SLEs) and incident chronic kidney disease (CKD) in middle-aged and older Chinese adults, as well as to examine frailty as a potential mediating pathway. **Methods**: We performed a prospective cohort study utilizing information from the 2011–2015 waves of the China Health and Retirement Longitudinal Study (CHARLS). SLEs were retrospectively assessed for childhood and adulthood. We utilized multivariable logistic regression models to determine the association between life-course SLEs and incident CKD. Subgroup analyses and interaction effects were used to detect possible differences across clinical and demographic subgroups. Mediation analyses were conducted to examine frailty’s role as a potential mediating pathway. Various sensitivity analyses were executed to test the primary outcomes’ stability. **Results**: In total, 3358 subjects were analyzed in the final study. During a 4-year follow-up, 241 individuals were detected with incident CKD. The fully adjusted model indicated that individuals who experienced SLE exposure were associated with higher odds of incident CKD than those who did not (OR = 2.05, 95% CI: 1.44–2.91, *p* < 0.001). When subjects were divided into four mutually exclusive SLE exposure patterns, those exposed to SLEs during both childhood and adulthood had the highest odds of incident CKD compared with those unexposed during either life stage (OR = 2.52, 95% CI: 1.66–3.81, *p* < 0.001), followed by those exposed only during adulthood (OR = 1.95, 95% CI: 1.30–2.93, *p* = 0.001) and those exposed only in childhood (OR = 1.83, 95% CI: 1.21–2.78, *p* = 0.004). The predefined subgroups exhibited no statistically significant interaction effects. Mediation analyses revealed that frailty statistically accounted for approximately 12% of the observed association between SLE exposure and incident CKD. **Conclusions**: Experiencing life-course SLEs was associated with higher odds of developing CKD in middle-aged and older Chinese adults. Frailty may represent one potential mediating pathway linking life-course SLEs with incident CKD. These results underscore the significance of considering life-course psychosocial stress and frailty when identifying individuals potentially vulnerable to CKD.

## 1. Introduction

Chronic kidney disease (CKD) poses a heavy public health burden worldwide [1], known for its extensive prevalence, insidious progression, and unfavourable outcomes [2]. As a frequent chronic illness around the world, CKD is closely associated with substantially elevated probability of developing end-stage kidney disease, cardiovascular disorders, and premature death [3,4]. As China’s population ages more rapidly, the incidence of CKD is increasing [5]. Nearly one-tenth of middle-aged and older Chinese adults have CKD, which significantly affects the socioeconomic well-being of these individuals, their families, and the country’s healthcare infrastructure [6,7]. Given the irreversible nature of advanced renal injury, early detection of modifiable risk factors for CKD is key to minimizing the public health burden. It is well recognized that the main contributors to CKD encompass hypertension, diabetes, dyslipidemia, obesity, and smoking [8,9]. In the past few years, growing data have shown a potential association between psychosocial stressors and the onset and advancement of CKD [10].

Stressful life events (SLEs) describe adverse experiences that may overwhelm a person’s coping capacity and elicit psychological and physiological stress responses [11]. These events can be classified according to the life stage during which they occur, namely as childhood SLEs or adulthood SLEs. Childhood SLEs encompass events such as parental unemployment, separating or divorcing, and parental death, among others [12]. Conversely, adulthood SLEs include experiences such as unemployment, losing a child, and losing a spouse or partner, among others [12]. A recent study has demonstrated a link between SLEs and the onset of CKD [13]; however, it should be noted that that study concentrated exclusively on SLEs experienced during adulthood. According to life-course theory, experiences throughout various life stages independently and interactively influence developmental trajectories and health outcomes [14]. Specifically, it is essential to explore the association of SLEs with kidney health from a life-course perspective. The childhood period constitutes a critical phase for organismal growth and development [15]. Childhood SLEs may be linked to long-term vulnerability to kidney disease through developmental, behavioural, and socioeconomic pathways. However, these associations may also be shaped by subsequent socioeconomic conditions, health behaviours, and chronic diseases during adulthood [16]. In contrast, adulthood SLEs may be associated with kidney dysfunction through sustained neuroendocrine, inflammatory, and behavioural pathways [17]. Experiencing stress in both childhood and adulthood can have cumulative adverse influences by combining early vulnerability with ongoing stress. Even so, longitudinal evidence is still limited concerning the relationship between CKD and SLE exposure occurring only in childhood, only in adulthood, or during both life stages.

Frailty is a geriatric syndrome reflecting multisystem physiological vulnerability [18]. High cumulative psychosocial stress is independently linked to frailty [19]. Furthermore, frailty is also prospectively linked to an elevated likelihood of incident CKD [20]. These findings collectively imply that frailty could be a possible mediating pathway between life-course SLEs and incident CKD. However, the extent to which frailty may explain this association remains unclear, especially among middle-aged and older Chinese adults.

To date, few longitudinal cohort studies have examined frailty as a potential mediating pathway linking life-course SLEs to incident CKD. Accordingly, our investigation leveraged the China Health and Retirement Longitudinal Study (CHARLS) data to investigate this association among middle-aged and older Chinese adults and evaluate effect modification across population subgroups alongside this possible mediating pathway.

## 2. Materials and Methods

### 2.1. Study Design and Population

This investigation relied on the CHARLS, a nationwide community-based prospective cohort study focusing on Chinese individuals who have reached the age of 45 and above. Baseline information (wave 1, 2011) was utilized to capture adulthood SLEs, sociodemographic and lifestyle factors, and comorbidities. Childhood SLEs were collected from the 2014 Life History Survey Questionnaire, and these events occurred prior to the 2011 baseline survey. CKD outcomes were ascertained at the 4-year follow-up wave (wave 3, 2015) after baseline.

Eligible individuals were those who (1) were at least 45 years old at baseline; (2) had no CKD at baseline (defined as no self-reported CKD and an estimated glomerular filtration rate (eGFR) of 60 mL/min/1.73 m^2^ or higher); and (3) provided complete self-reported data about childhood and adulthood SLEs.

**Figure 1:**
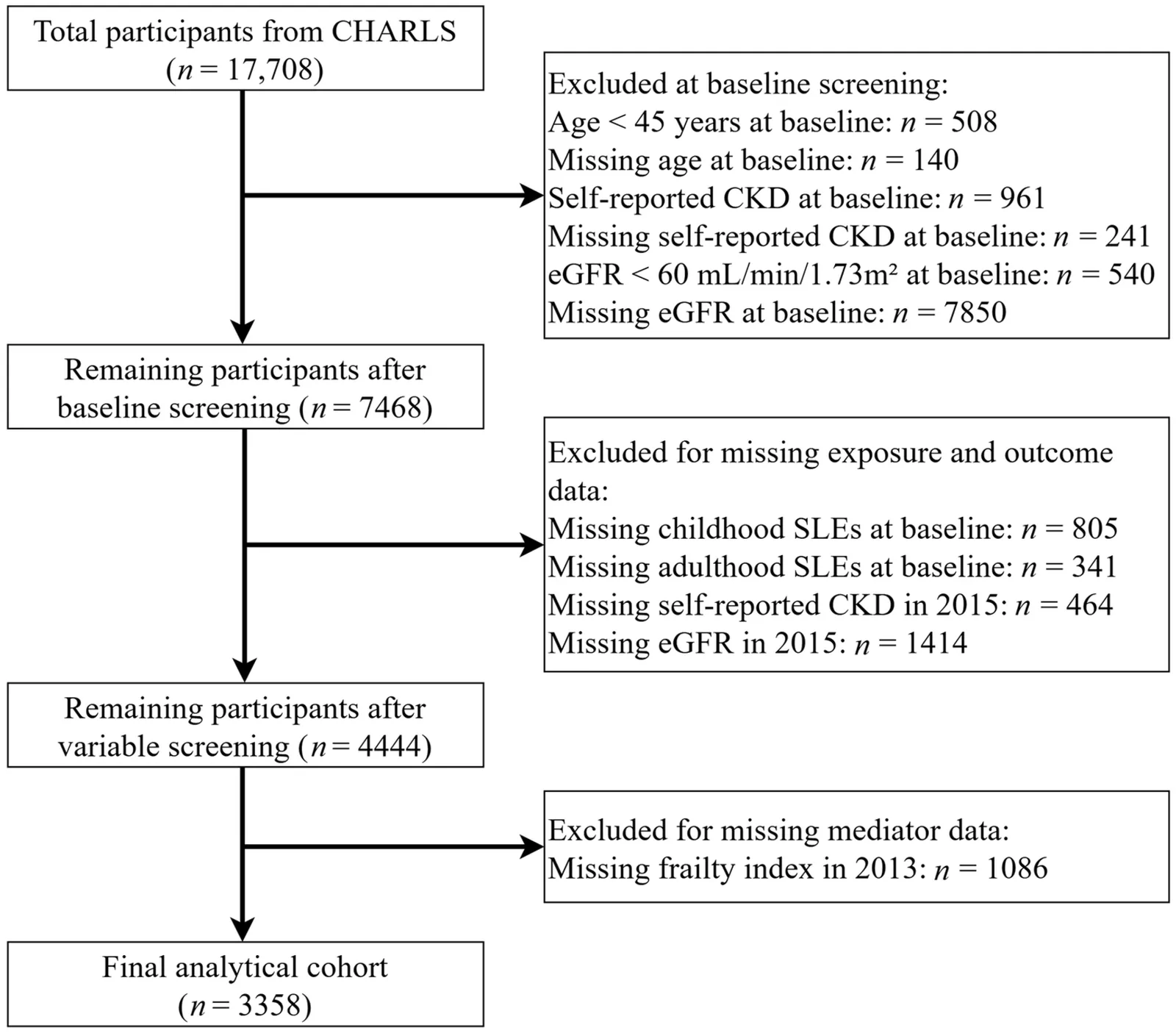
Flowchart of participant selection from the CHARLS cohort; 17,708 individuals were assessed for eligibility. During the baseline screening phase, 508 individuals were excluded for being under 45 years old, 140 for missing age data, 961 for self-reported chronic kidney disease (CKD), 241 for missing self-reported CKD information, 540 for having an estimated glomerular filtration rate (eGFR) below 60 mL/min/1.73 m^2^, and 7850 for missing eGFR data at baseline. In the subsequent variable screening phase, 805 individuals were excluded due to missing childhood stressful life event (SLE) data at baseline, 341 due to missing adulthood SLE data at baseline, 464 due to missing self-reported CKD information in 2015, and 1414 due to missing eGFR data in 2015. Additionally, 1086 were excluded for missing frailty index data in 2013, leaving a final sample of 3358 individuals included in the analysis. CHARLS, China Health and Retirement Longitudinal Study; CKD, chronic kidney disease; eGFR, estimated glomerular filtration rate; SLE, stressful life event. This figure was created by Figdraw 2.0 (www.figdraw.com) (ID: OPSRY921d2).

Individuals were not considered if their frailty index (FI) data were unavailable at the follow-up wave in 2013. Figure 1 details the flowchart for participant selection.

### 2.2. Stressful Life Events

Retrospectively self-reported SLEs were categorized into childhood and adulthood adverse experiences, in alignment with harmonized measurement frameworks utilized in previous CHARLS-based studies [12,21]. Childhood SLEs encompassed financial difficulties, parents being unemployed, parents abusing substances, being placed in foster care, parents getting separated or divorced, and losing a parent. Adulthood SLEs involved being unemployed, losing a child, losing a spouse or partner, experiencing a critical disease or incident, and being physically attacked or injured. Supplementary Table S1 contains the distribution of childhood and adulthood SLEs among the study participants. The count of SLEs experienced during childhood and adulthood was categorized as a categorical variable (0, 1, ≥2 events) to assess graded risk gradients, with reference to prior literature [12,13,22]. Based on previous studies [12,21], life-course SLE exposure was further divided into four groups: (1) absence of SLEs; (2) SLEs experienced solely in childhood; (3) SLEs experienced solely in adulthood; (4) SLEs experienced in both childhood and adulthood.

### 2.3. Incident CKD

Referring to previous studies [23,24,25,26,27], incident CKD was defined using laboratory-based eGFR assessments and self-reported physician-diagnosed kidney disease across two survey waves. At the baseline wave in 2011, participants were required to self-report an absence of prior physician-diagnosed renal disorder, and to have an eGFR of ≥60 mL/min/1.73 m^2^, assessed by the Chronic Kidney Disease Epidemiology Collaboration (CKD-EPI) (2021) creatinine–cystatin formula [28] $\mathrm{eGFR}\left(\mathrm{mL}/\min/1.73\;m^{2}\right)=135\times {\min\left(\frac{\mathrm{SCr}}{k},1\right)}^{\alpha }\times$ ${\max\left(\frac{\mathrm{SCr}}{k},1\right)}^{-0.544}\times {\min\left(\frac{\mathrm{SCys}}{0.8},1\right)}^{-0.323}\times {\max\left(\frac{\mathrm{SCys}}{0.8},1\right)}^{-0.778}\times 0.9961^{\mathrm{age}}\times 0.963\;(\mathrm{if female})$, where SCr is serum creatinine (mg/dL), SCys is serum cystatin C (mg/L), k is 0.7 for females and 0.9 for males, and α is −0.219 for females and −0.144 for males.

The emergence of new cases of CKD at the follow-up wave in 2015 was identified if participants satisfied either of the following criteria: (1) a follow-up eGFR below 60 mL/min/1.73 m^2^ or (2) reported receiving a diagnosis of a renal disorder from a physician during follow-up interviews. For robustness testing in the sensitivity analysis, an alternative outcome definition was employed, relying exclusively on self-reported physician-diagnosed renal disorder, to examine if the primary outcomes were altered by varying ascertainment strategies. This approach to define events of CKD was also already adopted in several CHARLS-based studies [29,30,31].

### 2.4. Frailty

Frailty in the 2013 follow-up wave was evaluated via an FI consisting of 32 distinct health deficits, with the full list of these 32 deficit items presented in Supplementary Table S2. The FI score was derived by taking the total number of identified health deficits, dividing it by 32, and then multiplying by 100, producing a continuous index from 0 to 100, where higher scores reflect a larger accumulation of health issues and increased frailty severity. Participants with an FI value ≥25 were categorized as frail for mediation analysis [32].

### 2.5. Covariates

A set of potential confounders was adjusted across regression models based on previous studies [33,34,35]. Demographic covariates included age and sex. Socioeconomic and lifestyle factors included region, marital status, education, smoking, drinking and body mass index (BMI). Medical history encompassed hypertension (defined as a systolic blood pressure (SBP) of 140 mmHg or higher, or a diastolic blood pressure (DBP) of 90 mmHg or higher, or a self-reported history of hypertension, or a self-reported history of using antihypertensive medications), diabetes (defined as a blood glucose level of 200 mg/dL or higher, or a fasting blood glucose (FBG) level of 126 mg/dL or higher, or a glycated hemoglobin A1c (HbA1c) level of 6.5% or higher, or a self-reported history of diabetes, or a self-reported history of using hypoglycemic medications), dyslipidemia (defined as a total cholesterol (TC) level of 240 mg/dL or higher, or a triglyceride (TG) level of 200 mg/dL or higher, or a low-density lipoprotein cholesterol (LDL-C) level of 160 mg/dL or higher, or a high-density lipoprotein cholesterol (HDL-C) level below 40 mg/dL, or a self-reported history of dyslipidemia, or a self-reported history of using lipid-lowering medications), and stroke (defined as a self-reported history of stroke, or a self-reported history of using stroke-related medications).

### 2.6. Statistical Analysis

Given the substantial number of excluded individuals, baseline demographic and clinical features were summarized for included and excluded participants to explore potential selection bias and attrition bias (Supplementary Table S3). Among those included, baseline characteristics were further stratified by SLE exposure, with weighted population-based descriptive statistics computed (Table 1). Survey sampling weights, stratification, and cluster-sampling parameters were not incorporated into any analyses in this study. We visually assessed the normality of all quantitative variables via quantile–quantile (Q-Q) plots, and found them to be distributed in a skewed manner (Supplementary Figures S1 and S2). For quantitative variables lacking a normal distribution, medians with interquartile ranges (IQRs) were used, while the representation of qualitative variables was in the form of numbers (percentages). The Mann–Whitney U test was employed to evaluate variations between groups for quantitative variables with skewed distribution, and the chi-square test was applied to investigate intergroup disparities in qualitative variables.

For covariates with missing proportions of less than 20%, single imputation with chained equations was utilized to deal with missing values. We adopted this 20% threshold consistent with previous CHARLS-based studies [36,37]. Continuous variables were handled using predictive mean matching, whereas dichotomous variables were analyzed with binary logistic regression. One completed dataset was generated (m = 1) using a prespecified random seed and 10 iterations. Age and sex were included as predictors within the imputation model. Little’s test rejected the assumption that the data were missing completely at random (MCAR) (χ^2^ = 291.15, df = 147, *p* < 0.001). Therefore, the imputation procedure was implemented under a missing-at-random assumption, conditional on the measured variables incorporated into the imputation models. The percentages of missing data for individual covariates are presented in Supplementary Table S4.

The development of progressively adjusted models aimed to compute odds ratios (ORs) and 95% confidence intervals (CIs) for incident CKD associated with SLE exposure: the Crude model was unadjusted for any covariates; Model 1 included adjustments for age and sex; Model 2 added adjustments for region, marital status, education, smoking, drinking, and BMI; and Model 3 further adjusted for hypertension, diabetes, dyslipidemia, and stroke.

Generalized variance inflation factors (GVIF) were calculated for all adjusted covariates to evaluate multicollinearity. We further computed the standardized index GVIF^1/(2*Df)^ for each confounder, where Df denotes the degrees of freedom of the corresponding covariate (Supplementary Tables S5 and S6). A value of GVIF^1/(2*Df)^ less than the conventional cutoff of 2 indicated negligible multicollinearity [38].

Referring to previous research [33], multiple subgroups were determined for subgroup analyses, including age, sex, education, smoking, drinking, hypertension, diabetes, and dyslipidemia. Considering that cognitive disorder and depression may influence the recall of SLE exposure and induce recall bias, we additionally performed subgroup analyses stratified by depression and cognitive disorder. Interaction terms were incorporated to evaluate effect modification.

Mediation analyses were used to divide the total effects of SLE exposure into direct effects (unmediated by frailty) and indirect effects (mediated through frailty). Given the chronological sequence of data collection (SLE exposure before 2011, frailty status assessed in 2013, and incident CKD identified in 2015) and the binary form of the outcome variable, exploratory mediation analysis based on the counterfactual estimation framework was executed utilizing the mediation package in R. The binary mediator variable was modelled via generalized linear regression, and the binary outcome variable was modelled utilizing logistic regression. Quasi-Bayesian Monte Carlo simulation with 1000 iterations was applied to estimate the average causal mediation effect (ACME, indirect effect), average direct effect (ADE, direct effect), and total effect. For binary outcomes, effect estimates (ACME, ADE, and total effect) generated by this package represent risk differences (RD) in the probability of outcome occurrence rather than log odds. The proportion of the total effect mediated by frailty was calculated as the ratio of ACME to the total effect. E-value sensitivity analyses were additionally conducted for the total and direct associations to assess their robustness to potential unmeasured confounding. On the risk-difference scale, the E-value estimates the smallest association strength required for an unmeasured confounder, with links to both exposure and outcome, to completely eliminate the detected association.

The main outcomes’ consistency was assessed through four distinct sensitivity analyses: (1) refitting the primary logistic regression model using the complete-case non-imputed dataset; (2) refitting the primary logistic regression model with an alternative incident CKD definition derived from self-reported doctor diagnosis; (3) refitting the primary logistic regression model in a restricted analytical sample excluding participants with baseline malignant tumours; (4) performing an unadjusted mediation analysis.

All statistical analyses employed R version 4.4.0 (24 April 2024) alongside Zstats 1.0 (www.zstats.net). A two-tailed *p* value below 0.05 indicated statistical significance. We reported this prospective cohort study following the Strengthening the Reporting of Observational Studies in Epidemiology (STROBE) guidelines for observational cohort studies.

## 3. Results

### 3.1. Study Population and Baseline Characteristics

The final analytical group incorporated 3358 participants in line with the inclusion criteria. Of these, 2225 individuals (66.3%) reported exposure to SLEs, whereas 1133 individuals (33.7%) reported no such exposure. As illustrated in Table 1, participants exposed to SLEs were significantly older and had higher odds of being unmarried, having lower BMI, a having higher rate of smoking, and having an increased occurrence of hypertension and stroke (all *p* < 0.05). Furthermore, the geographic distribution and educational attainment differed significantly between participants with and without SLE exposure (both *p* < 0.05). No notable intergroup differences were detected for sex distribution, alcohol consumption, diabetes, or dyslipidemia (all *p* > 0.05). Baseline characteristics of enrolled versus excluded individuals are presented in Supplementary Table S3. Excluded participants had lower odds of being married, lower BMI, higher stroke prevalence, and lower prevalence of hypertension, diabetes and dyslipidemia, and differed in educational distribution compared with those included (all *p* < 0.05).

### 3.2. Association Between SLEs and Incident CKD

Significant associations were found between incident CKD and both categorical SLE exposure and cumulative SLE counts (Table 2). After controlling for potential confounding factors, participants exposed to SLEs during either childhood or adulthood had higher odds of incident CKD than those without SLE exposure (fully adjusted OR = 2.05, 95% CI: 1.44–2.91, *p* < 0.001). Each additional SLE was also linked to higher odds of incident CKD (fully adjusted OR = 1.31, 95% CI: 1.15–1.49, *p* < 0.001).

When SLEs were further divided into childhood and adulthood adverse experiences, the association remained significant. Compared to individuals without SLEs, those exposed to SLEs during adulthood had higher odds of incident CKD (fully adjusted OR = 1.63, 95% CI: 1.22–2.18, *p* < 0.001), with childhood SLEs following closely (fully adjusted OR = 1.50, 95% CI: 1.14–1.97, *p* = 0.004). Additionally, multicollinearity assessments were conducted both with and without FI included as a model covariate; no substantial collinearity was identified under either specification (Supplementary Tables S5 and S6).

We then reclassified life-course SLEs into four mutually exclusive subgroups to disentangle separate and combined exposure effects (Table 3). In comparison to participants who reported no exposure to SLEs, exposure to SLEs during both childhood and adulthood exhibited the greatest odds of incident CKD (fully adjusted OR = 2.52, 95% CI: 1.66–3.81, *p* < 0.001). This was followed by exposure occurring exclusively in adulthood (fully adjusted OR = 1.95, 95% CI: 1.30–2.93, *p* = 0.001) and childhood (fully adjusted OR = 1.83, 95% CI: 1.21–2.78, *p* = 0.004).

### 3.3. Subgroup Analyses and Interaction Tests

**Figure 2:**
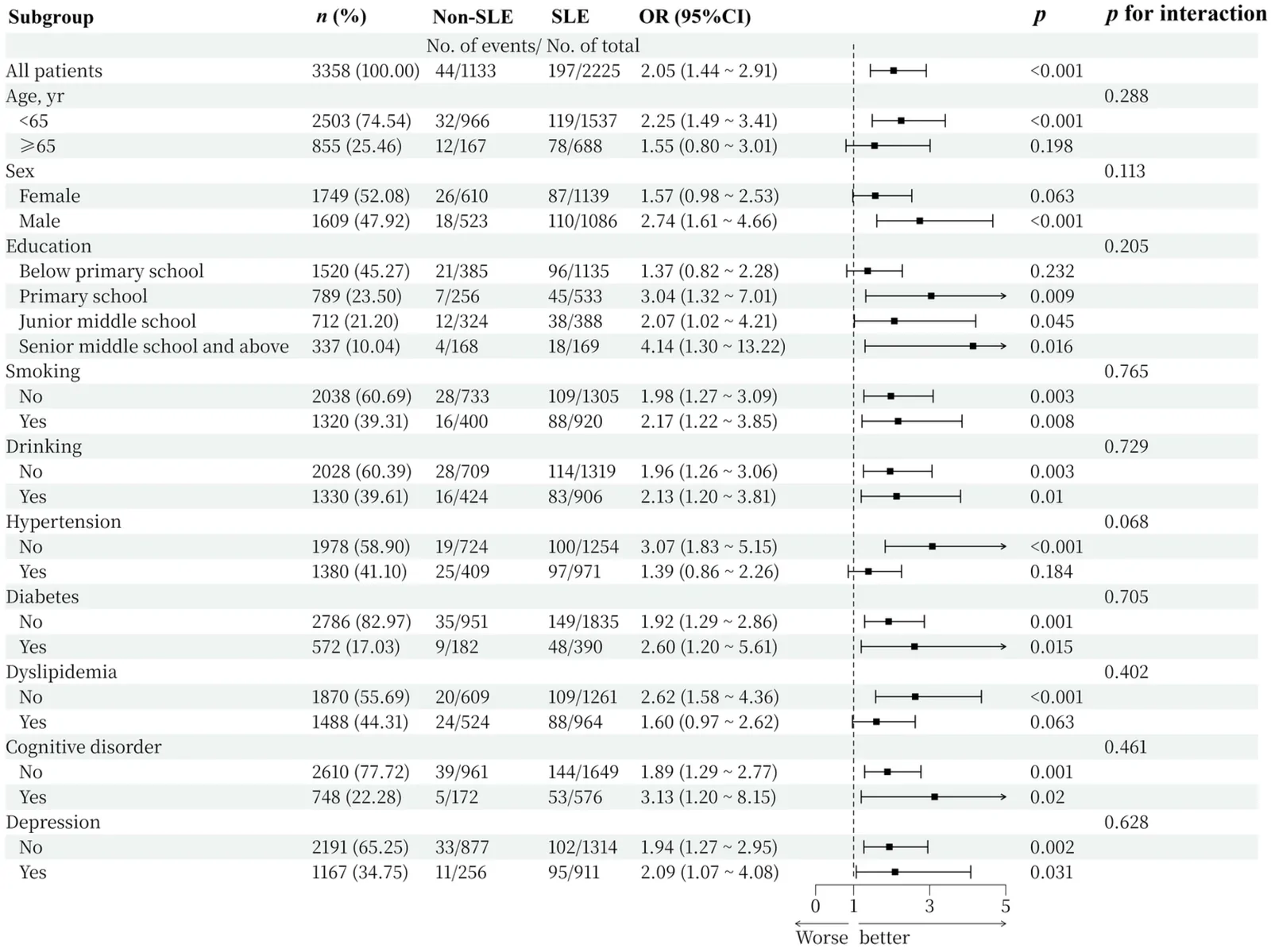
Forest plot for subgroup analyses of the association between stressful life events and incident chronic kidney disease. SLE, stressful life event; OR, odds ratio; CI, confidence interval.

Subgroup analyses by demographic and clinical characteristics are presented in Figure 2. The magnitude of the association between SLE exposure and incident CKD was visualized across different subgroups. No statistically supported effect modification was identified within the examined subgroups (all *p* for interaction > 0.05).

### 3.4. Mediation Analyses

**Figure 3:**
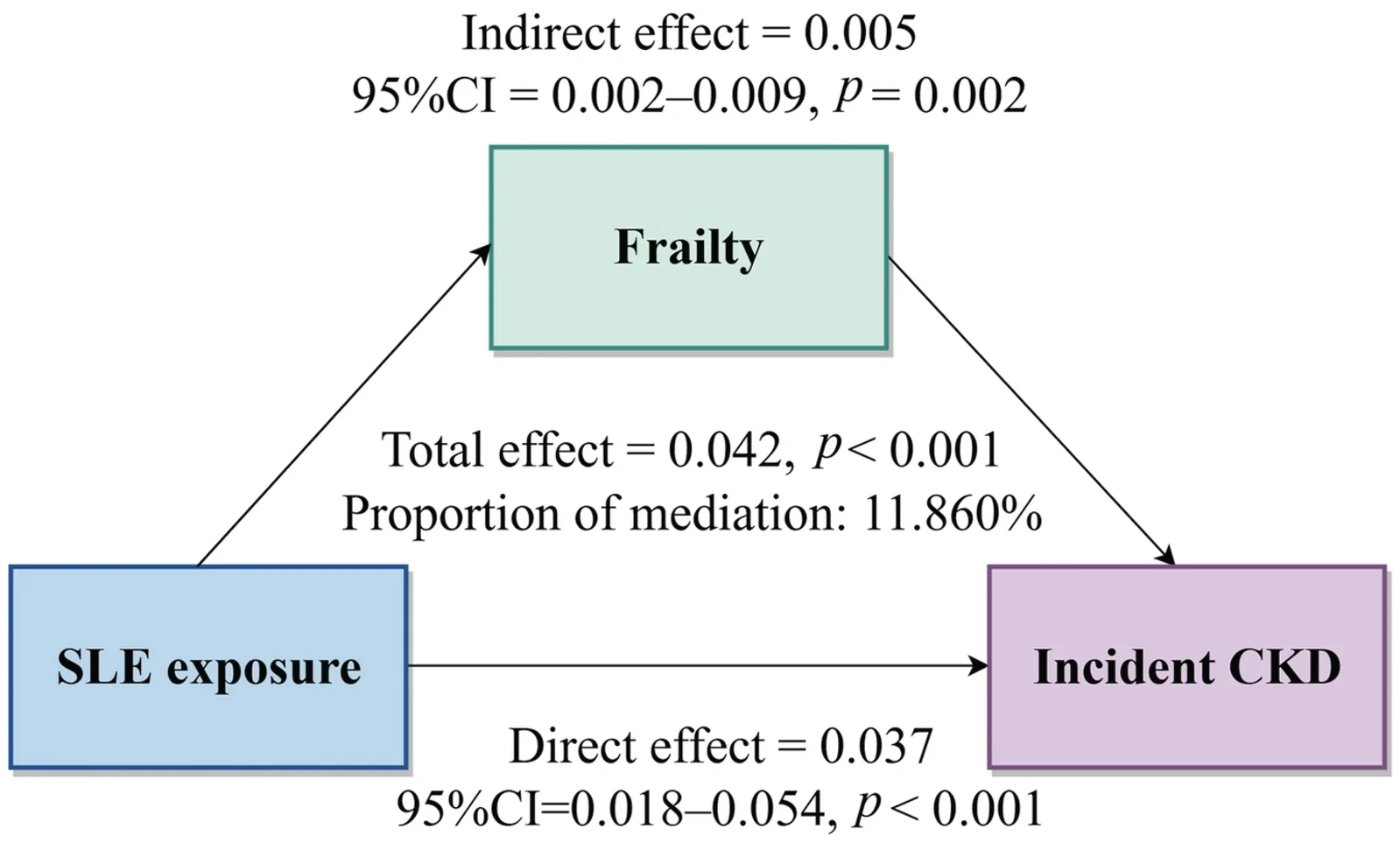
Illustration of mediation effect. CKD, chronic kidney disease; SLE, stressful life event. This figure was created by Figdraw 2.0 (www.figdraw.com) (ID: IPAUS40650).

The total effect of SLE exposure on incident CKD was 0.042 (95% CI: 0.024–0.059, *p* < 0.001), with an E-value of 1.255 and an E-value of 1.181 calculated for the 95% CI limit closest to the null. Both the ADE (direct effect) and ACME (indirect effect) of SLE exposure on incident CKD were statistically significant. The ADE was 0.037 (95% CI: 0.018–0.054; *p* < 0.001), with an E-value of 1.24 for the point estimate and an E-value of 1.15 for the 95% CI limit closest to the null. The ACME was 0.005 (95% CI: 0.002–0.009, *p* = 0.002). Frailty statistically accounted for 11.860% of the observed association between SLE exposure and incident CKD (Figure 3).

### 3.5. Sensitivity Analyses

To assess the reliability of the core results, four independent sensitivity analyses were conducted from distinct methodological perspectives. First, we re-estimated the primary association within the complete-case dataset without imputation of missing values. After full multivariable adjustment, SLE exposure remained significantly linked to higher odds of incident CKD (OR = 1.85, 95% CI: 1.26–2.72, *p* = 0.002; Supplementary Table S7). Second, we adopted an alternative definition of incident CKD relying on self-reported physician diagnosis to test whether our core findings were sensitive to varying outcome ascertainment criteria, and the statistical significance of effect estimates was largely unchanged (Supplementary Table S8). However, this sensitivity analysis should be interpreted with caution, as self-reported CKD may have limited sensitivity, particularly for early-stage CKD, which tends to be asymptomatic and might not be diagnosed. Third, we excluded all participants with baseline cancer to mitigate potential residual confounding induced by malignant disease, and the robust positive relationship between SLE exposure and incident CKD was still observed in the restricted analytical sample (Supplementary Table S9). Fourth, we performed an unadjusted mediation analysis to evaluate whether the overlap between comorbidity items in the FI and baseline covariates would alter the mediating estimates. The mediating estimates were broadly consistent with the fully adjusted model, suggesting the observed potential mediating pathway remained stable despite this item overlap (Supplementary Figure S3). Throughout the four sensitivity tests, the association’s direction and statistical significance were consistent with the primary analysis, reinforcing the dependability of our primary results.

## 4. Discussion

Our investigation found that life-course SLEs were associated with heightened odds of incident CKD among middle-aged and older Chinese adults. Subgroup analysis revealed no statistical evidence of effect modification across the examined subgroups. Additionally, the core results remained largely consistent through multiple sensitivity analyses. Mediation analysis indicated that frailty may explain approximately 12% of the association between life-course SLEs and incident CKD.

CKD is a complex disorder characterized by multiple risk factors, and its prevention and treatment remain suboptimal [39]. A previous study by Luo et al. analyzed data from 10,247 individuals in the CHARLS [40]. The results indicated that adverse childhood experiences—such as physical abuse, residing in a dangerous area, having a disabled parent, and others—were associated with higher odds of developing CKD. Another investigation revealed that experiencing negative life events from childhood to adulthood, including childhood abuse, adulthood adversity, and severe trauma, was associated with a heightened risk of incident CKD [41]. Collectively, these studies support an association between psychosocial adversity and adverse kidney health.

SLEs have been proposed as potential psychosocial correlates of adverse health outcomes. A prior investigation revealed a notable connection between childhood SLEs and an increased probability of stroke within two national cohorts [33]. In a distinct study involving participants from three national cohorts, findings demonstrated an association between adulthood SLEs and the onset of heart disease [42]. A newly published study found that adulthood SLEs were also associated with incident CKD [13]. Nevertheless, the majority of previous studies have examined either childhood or adulthood SLEs independently, without considering the combined effects across the life course. Therefore, we expanded the research by examining the relationship of SLE exposure occurring solely in childhood, solely in adulthood, and in both childhood and adulthood with incident CKD. Our findings further suggest that the link between SLE exposure throughout childhood and adulthood and incident CKD was more pronounced than that observed for SLEs occurring exclusively in childhood or adulthood, corroborating previous research [12,43]. Zhou et al. analyzed longitudinal datasets from three cohorts, reporting risk estimates of 1.32 (95% CI: 1.19–1.48), 1.44 (95% CI: 1.30–1.59), and 1.89 (95% CI: 1.69–2.11) for physical–psychological–cognitive multimorbidity in individuals exposed to SLEs solely in childhood, solely in adulthood, and in both childhood and adulthood, respectively [12]. Another recent study also confirmed that the detrimental effects on intrinsic abilities resulting from SLEs experienced in both childhood and adulthood were significantly greater than those caused by exposure to SLEs during only one of these life stages [43]. Two major life-course frameworks may help interpret this observed risk gradient: the cumulative-stress hypothesis and the stress-sensitization hypothesis [44]. Under the cumulative-stress framework, each SLE incrementally contributes to total psychosocial burden; individuals experiencing SLEs in both periods accrue the greatest total stress burden, resulting in elevated disease risk [44]. Alternatively, the stress-sensitization model posits that childhood adversity may permanently alter neuroendocrine, inflammatory, and metabolic regulatory systems, rendering organisms more vulnerable to the pathological impacts of subsequent stressors in adulthood, even if later-life stress events themselves are modest in magnitude [45]. These results underscore the necessity of considering a life-course perspective to gain a deeper insight into the psychosocial contributors to CKD risk.

There are several plausible, literature-supported pathways that might underpin the association between life-course SLEs and incident CKD. From a neuroendocrine perspective, prolonged exposure to SLEs can drive over-activation of the sympathetic nervous system and dysregulation of the hypothalamic–pituitary–adrenal (HPA) axis [17]. Sustained sympathetic hyperactivity promotes renal vasoconstriction, glomerular hyperfiltration injury, podocyte foot process effacement, and renal microvascular endothelial damage [46]; concurrently, sympathetic over-stimulation triggers excessive renin–angiotensin–aldosterone system (RAAS) activation, further amplifying intraglomerular hypertension and perpetuating renal parenchymal injury [17,47]. Early-life adverse stress in particular may induce long-term HPA-axis set-point shifts, leading to aberrant diurnal cortisol secretion patterns [17]. Such neuroendocrine perturbation sustains elevations of pro-inflammatory mediators including interleukin-6 (IL-6), tumour necrosis factor-α (TNF-α), and high-sensitivity C-reactive protein (hs-CRP) over decades [17,48]. Persistent low-grade systemic inflammation in turn accelerates systemic vascular senescence and propagates tubulointerstitial fibrosis, two hallmark pathological processes driving progressive CKD. Chronic stress-related oxidative stress acts synergistically with inflammatory signalling to exacerbate endothelial dysfunction within the renal microcirculation, further hastening renal function decline [17,49]. Beyond biological pathways, behavioural and socioeconomic mechanisms may also link SLE exposure to incident CKD. Individuals experiencing SLEs are more prone to modifiable unhealthy behaviours including tobacco use, suboptimal dietary patterns, and low physical activity [50,51], all well-established risk factors for kidney impairment [52,53,54]. Socioeconomic disadvantage accompanying many SLEs may limit access to healthcare resources and impair long-term control of cardiometabolic comorbidities such as hypertension and diabetes, which are major upstream drivers of CKD progression [55,56].

Further investigations of this potential mediating pathway may improve our understanding of the association between SLE exposure and incident CKD. Previous studies have associated SLE exposure with frailty and frailty with incident CKD [57,58,59]. Reyes-Ortiz and colleagues reviewed fifteen studies published from 2018 to 2025, examining the connection between adverse childhood experiences, SLEs, and frailty among older adults [57]. Their findings indicated a significant correlation between adverse childhood experiences, SLEs, and other adversity measures with an increased likelihood of frailty occurrence. In another investigation by Zhang et al., involving 3597 participants from the CHARLS, it was observed that individuals with pre-frailty or frailty were more prone to developing CKD than robust individuals [58]. Moreover, the research by Qu and coworkers analyzed data from both CHARLS (N = 7013) and the UK Biobank (N = 451,067), also revealing a notable association between frailty and the likelihood of developing CKD in both cohorts [59]. Consequently, we further explored the role of frailty as a potential mediating pathway in the association between life-course SLEs and incident CKD, and found suggestive evidence of partial mediation.

Frailty, a common condition in older adults, is identified by declining physiological reserves and increasing susceptibility to stress [60]. The plausible explanations supported by previous literature for how frailty may act as a potential mediating pathway in the association between life-course SLEs and incident CKD can be delineated as follows. On the one hand, chronic stress might disrupt the homeostasis of the HPA axis, potentially altering the normal secretion pattern of cortisol [61,62]. Previous research has indicated that reduced morning and elevated evening salivary cortisol levels are correlated with frailty [63,64]. On the other hand, when considering the link between frailty and CKD, diminished physical activity, indicative of frailty, may heighten the risk of developing CKD [65,66].

This study features the following strengths. Firstly, as far as we know, this is one of the initial investigations into the link between life-course SLEs and incident CKD among middle-aged and older Chinese adults from the viewpoint of a life course. Secondly, this investigation made use of information from CHARLS, a major cohort study in China renowned for its national representativeness and longitudinal follow-up, which improves the generalizability of the study’s results. Thirdly, this research accounted for a range of confounding variables, including demographic characteristics, behavioural factors, and disease burden, to support the robustness of the results. Fourthly, our findings showed a high level of consistency in multiple sensitivity and subgroup analyses. Lastly, our findings hold significant public health importance and are applicable to the middle-aged and older Chinese adults. Beyond the conventional metabolic and cardiovascular risk factors, psychosocial stress and frailty deserve attention from clinicians. In clinical practice, general practitioners and nephrologists may adopt brief screening instruments for psychosocial stress and frailty. Those with positive screening results can be prioritized for renal function and urine protein testing to identify individuals who may gain benefits from early kidney protective strategies.

Our investigation has certain constraints that warrant acknowledgment. Firstly, childhood SLEs were retrospectively reported by middle-aged or older adults at the time of assessment, and recall of these experiences may be influenced by age, current socioeconomic status, psychological state, cognitive function, and health status, which may introduce recall bias and reporting bias. On the one hand, differential recall bias may lead to overreporting of childhood SLEs, which could overestimate the magnitude of the observed association. For example, patients with CKD may exhibit mood-congruent recall, which could inflate their reporting of early-life adversities. On the other hand, nondifferential memory decay over decades may introduce random measurement error, which tends to attenuate the magnitude of association toward the null hypothesis. Secondly, selective survival bias merits consideration. Given that CHARLS targets middle-aged and older adults, individuals with severe childhood stress and biological vulnerability may have died or developed end-stage kidney disease or severe cardiovascular disease before the 2011 baseline survey. Accordingly, exposed survivors enrolled in the cohort may constitute a resilient subgroup, which could underestimate the true association and may even reverse the direction of the observed relationship. In addition, exclusion of participants due to missing data may introduce further selection bias. Attrition during follow-up may also bias the association if loss to follow-up is related to both life-course SLE exposure and kidney health status. Although CHARLS was designed to be nationally representative, the markedly reduced analytical sample may not fully preserve this representativeness, potentially limiting the external validity of our findings. Therefore, future prospective longitudinal studies collecting exposure data from children or young adults at an earlier life stage are needed to further validate these findings. Thirdly, merely counting SLEs may not accurately capture an individual’s actual stress burden. This metric only quantifies the number of reported adverse events, without capturing event severity, duration, frequency, timing, or subjective appraisal. Therefore, this indicator should not be regarded as a precise quantitative measure of cumulative psychosocial stress burden. Fourthly, misclassification of renal outcomes may exist. CHARLS only provides eGFR data from the 2011 and 2015 waves, with no eGFR measurements available in 2013. Therefore, it remains unclear whether health deficits captured at the 2013 frailty assessment included renal decline occurring prior to the 2015 CKD ascertainment. Lack of urinalysis, renal imaging, and renal pathology data restricts our composite CKD definition to limited repeated renal function measurements and self-reported physician diagnoses. This prevents strict adherence to Kidney Disease: Improving Global Outcomes (KDIGO) guidelines [67] and restricts our ability to confirm persistent renal impairment. In addition, eGFR available only at these two waves prevents determination of the exact timing of incident CKD onset. Time-to-event survival analyses including Cox and competing-risk models were therefore not feasible. We used binary logistic regression for 4-year cumulative CKD incidence, which ignores event timing, censoring, and competing mortality. Fifthly, potential residual confounding cannot be fully ruled out, as several variables representing known or unknown kidney-related risk factors were unavailable in CHARLS, including thyroid status [68], systemic inflammation [69], family history of kidney failure [70], dietary sodium and potassium intake [71], medication exposure (e.g., corticosteroids [72], non-steroidal anti-inflammatory drugs (NSAIDs) [73], aminoglycosides [74], proton pump inhibitors [75], and RAAS blockers [76]), and key socioeconomic indicators such as household income, occupation, residence, childhood socioeconomic circumstances, and healthcare access [77]. Sixthly, we are unable to disentangle competing explanations for non-significant subgroup associations. Wide confidence intervals suggest limited statistical power, probably due to fewer outcome events in these subgroups. Competing mortality risk among older adults and genuine biological resilience may also affect the results, though we cannot separately assess these possibilities. Future larger cohorts with adequate outcome events and competing-risk survival models may help separate the effects of limited statistical power and competing mortality. Seventhly, this exploratory mediation analysis relies on the untestable sequential ignorability assumption. Although we conducted E-value sensitivity analysis to assess potential unmeasured confounding, residual confounding cannot be fully excluded, and the identified mediating pathway should therefore be interpreted cautiously rather than as definitive causal evidence. In addition, the 2013 frailty assessment may capture pre-existing health deficits already present at the 2011 baseline. Baseline frailty was not adjusted for, as it lies on the causal pathway between life-course SLEs and the 2013 frailty status; adjusting for this intermediate variable would block the pathway of interest. Furthermore, several fully adjusted covariates (hypertension, diabetes, BMI, and smoking) may potentially lie on the pathway linking SLEs, frailty, and CKD. Due to insufficient temporal information in the CHARLS dataset, we could not unambiguously classify these variables as confounders or intermediates, and therefore did not construct a directed acyclic graph (DAG) to define causal structure. This uncertainty in covariate classification represents an inherent limitation of the present exploratory analysis. Moreover, some items of the 32-deficit frailty index conceptually overlap with baseline comorbidity covariates (e.g., stroke and metabolic disorders) in the mediation models, raising concerns about conceptual circularity. While GVIF tests ruled out severe statistical collinearity, this conceptual overlap remains a limitation. Eighthly, ORs derived from logistic regression should be interpreted with caution in this prospective cohort setting. ORs will overestimate risk ratio (RR) when the incidence of the outcome is non-negligible; thus, OR values in the present study cannot be directly interpreted as RRs for incident CKD. Ninthly, missing covariate data were addressed using single imputation, which does not fully account for uncertainty associated with the imputed values and may consequently underestimate standard errors. Furthermore, only age and sex were incorporated as predictors in the imputation model; this parsimonious set of predictors may not fully capture information associated with missingness, potentially limiting the performance of the imputation. In addition, Little’s MCAR test suggested that the data were unlikely to be missing completely at random; therefore, bias related to the missing-data mechanism cannot be entirely excluded. Nevertheless, the findings from the complete-case analysis were generally consistent with the primary results. Tenthly, reverse causation also merits consideration. Subclinical renal impairment or early kidney damage may precede and worsen frailty and increase vulnerability to psychosocial stress, which could partially explain the observed association. Lastly, the applicability of these conclusions is restricted to Chinese individuals over the age of 45 and might not be applicable to younger individuals or non-Chinese populations.

Our future efforts will focus on the following directions. Firstly, it is imperative to incorporate a more diverse cohort, encompassing individuals from a range of countries and ethnic backgrounds, to validate our findings. Secondly, the implementation of long-term cohort studies, which include repeated assessments of kidney function, is essential for conducting more in-depth analyses. Thirdly, acquiring more detailed and comprehensive data on SLEs is vital for investigating how the severity and duration of SLE exposure relate to incident CKD.

## 5. Conclusions

In conclusion, life-course exposure to SLEs was associated with higher odds of incident CKD among middle-aged and older Chinese adults. The association was strongest among individuals exposed to SLEs during both childhood and adulthood. Frailty may represent one potential pathway linking life-course SLEs with subsequent CKD. Further longitudinal studies with repeated assessments of kidney function and more comprehensive measures of stress are warranted.

## Figures and Tables

**Table 1:** Baseline characteristics of participants depending on stressful life event exposure.

Variables	Total (*n* = 3358)	Stressful Life Event Exposure		*p*	SMD
		No (*n* = 1133)	Yes (*n* = 2225)		
Age, year, Median (Q_1_, Q_3_)	58.00 (52.00, 65.00)	56.00 (50.00, 61.00)	60.00 (54.00, 66.00)	<0.001 *	0.461
BMI, kg/m^2^, Median (Q_1_, Q_3_)	23.33 (21.09, 26.09)	23.85 (21.54, 26.39)	23.12 (20.84, 25.87)	<0.001 *	0.150
Sex, *n* (%)				0.146	0.053
Female	1749 (52.08)	610 (53.84)	1139 (51.19)		
Male	1609 (47.92)	523 (46.16)	1086 (48.81)		
Region, *n* (%)				<0.001 *	0.147
Eastern China	1521 (45.29)	568 (50.13)	953 (42.83)		
Central China	545 (16.23)	189 (16.68)	356 (16.00)		
Western China	1045 (31.12)	291 (25.68)	754 (33.89)		
Northeast China	247 (7.36)	85 (7.50)	162 (7.28)		
Marital status, *n* (%)				<0.001 *	0.547
Married	3014 (89.76)	1124 (99.21)	1890 (84.94)		
Other	344 (10.24)	9 (0.79)	335 (15.06)		
Education, *n* (%)				<0.001 *	0.415
Below primary school	1520 (45.27)	385 (33.98)	1135 (51.01)		
Primary school	789 (23.50)	256 (22.59)	533 (23.96)		
Junior middle school	712 (21.20)	324 (28.60)	388 (17.44)		
Senior middle school and above	337 (10.04)	168 (14.83)	169 (7.60)		
Smoking, *n* (%)				<0.001 *	0.125
No	2038 (60.69)	733 (64.70)	1305 (58.65)		
Yes	1320 (39.31)	400 (35.30)	920 (41.35)		
Drinking, *n* (%)				0.065	0.068
No	2028 (60.39)	709 (62.58)	1319 (59.28)		
Yes	1330 (39.61)	424 (37.42)	906 (40.72)		
Hypertension, *n* (%)				<0.001 *	0.154
No	1978 (58.90)	724 (63.90)	1254 (56.36)		
Yes	1380 (41.10)	409 (36.10)	971 (43.64)		
Diabetes, *n* (%)				0.286	0.039
No	2786 (82.97)	951 (83.94)	1835 (82.47)		
Yes	572 (17.03)	182 (16.06)	390 (17.53)		
Dyslipidemia, *n* (%)				0.107	0.059
No	1870 (55.69)	609 (53.75)	1261 (56.67)		
Yes	1488 (44.31)	524 (46.25)	964 (43.33)		
Stroke, *n* (%)				<0.001 *	0.151
No	3287 (97.89)	1124 (99.21)	2163 (97.21)		
Yes	71 (2.11)	9 (0.79)	62 (2.79)		

Q_1_, 1st quartile; Q_3_, 3rd quartile; SMD, Standardized Mean Differences; * *p* < 0.05.

**Table 2:** Association between stressful life event exposure and incident chronic kidney disease.

Variables	Events/Total, *n*	Crude Model		Model 1		Model 2		Model 3	
		OR (95%CI)	*p*	OR (95%CI)	*p*	OR (95%CI)	*p*	OR (95%CI)	*p*
The pooled population	241/3358								
Stressful life event exposure									
No	44/1133	1.00 (Reference)		1.00 (Reference)		1.00 (Reference)		1.00 (Reference)	
Yes	197/2225	2.40 (1.72, 3.36)	<0.001 *	2.11 (1.50, 2.97)	<0.001 *	2.12 (1.49, 3.00)	<0.001 *	2.05 (1.44, 2.91)	<0.001 *
Count of stressful life events									
Per stressful life event		1.37 (1.22, 1.53)	<0.001 *	1.28 (1.14, 1.44)	<0.001 *	1.31 (1.16, 1.49)	<0.001 *	1.31 (1.15, 1.49)	<0.001 *
0	44/1133	1.00 (Reference)		1.00 (Reference)		1.00 (Reference)		1.00 (Reference)	
1	89/1188	2.00 (1.38, 2.90)	<0.001 *	1.85 (1.27, 2.68)	0.001 *	1.84 (1.26, 2.68)	0.002 *	1.78 (1.22, 2.60)	0.003 *
≥2	108/1037	2.88 (2.00, 4.13)	<0.001 *	2.44 (1.68, 3.53)	<0.001 *	2.56 (1.74, 3.77)	<0.001 *	2.47 (1.68, 3.65)	<0.001 *
*p* for trend		<0.001 *		<0.001 *		0.001 *		0.002 *	
Childhood stressful life event exposure									
No	113/1954	1.00 (Reference)		1.00 (Reference)		1.00 (Reference)		1.00 (Reference)	
Yes	128/1404	1.63 (1.26, 2.13)	<0.001 *	1.52 (1.16, 1.98)	0.002 *	1.50 (1.14, 1.96)	0.004 *	1.50 (1.14, 1.97)	0.004 *
Count of childhood stressful life events									
Per stressful life event		1.40 (1.18, 1.67)	<0.001 *	1.33 (1.12, 1.59)	0.002 *	1.32 (1.10, 1.59)	0.003 *	1.33 (1.10, 1.60)	0.003 *
0	113/1954	1.00 (Reference)		1.00 (Reference)		1.00 (Reference)		1.00 (Reference)	
1	94/1099	1.52 (1.15, 2.02)	0.004 *	1.43 (1.07, 1.90)	0.015 *	1.41 (1.05, 1.88)	0.021 *	1.41 (1.06, 1.89)	0.020 *
≥2	34/305	2.04 (1.36, 3.06)	<0.001 *	1.85 (1.23, 2.79)	0.003 *	1.82 (1.20, 2.76)	0.005 *	1.82 (1.20, 2.76)	0.005 *
*p* for trend		<0.001 *		<0.001 *		0.001 *		0.002 *	
Adulthood stressful life event exposure									
No	97/1846	1.00 (Reference)		1.00 (Reference)		1.00 (Reference)		1.00 (Reference)	
Yes	144/1512	1.90 (1.45, 2.48)	<0.001 *	1.65 (1.26, 2.18)	<0.001 *	1.70 (1.27, 2.27)	<0.001 *	1.63 (1.22, 2.18)	<0.001 *
Count of adulthood stressful life events									
Per stressful life event		1.42 (1.21, 1.67)	<0.001 *	1.27 (1.08, 1.51)	0.005 *	1.34 (1.11, 1.61)	0.002 *	1.31 (1.08, 1.58)	0.005 *
0	97/1846	1.00 (Reference)		1.00 (Reference)		1.00 (Reference)		1.00 (Reference)	
1	102/1118	1.81 (1.36, 2.42)	<0.001 *	1.64 (1.22, 2.19)	0.001 *	1.65 (1.22, 2.23)	0.001 *	1.59 (1.17, 2.15)	0.003 *
≥2	42/394	2.15 (1.47, 3.15)	<0.001 *	1.71 (1.15, 2.56)	0.008 *	1.89 (1.23, 2.91)	0.004 *	1.82 (1.18, 2.81)	0.007 *
*p* for trend		<0.001 *		<0.001 *		<0.001 *		0.001 *	

OR, odds ratio; CI, confidence interval; * *p* < 0.05. Crude model: without adjustments (no covariates included). Model 1: adjustments made for age and sex. Model 2: further adjustments for region, marital status, education, smoking, drinking, and body mass index (BMI). Model 3: additional adjustments for hypertension, diabetes, dyslipidemia, and stroke.

**Table 3:** The association between four-category stressful life event exposure and incident chronic kidney disease at follow-up.

Variables	Events/Total, *n*	Crude Model		Model 1		Model 2		Model 3	
		OR (95%CI)	*p*	OR (95%CI)	*p*	OR (95%CI)	*p*	OR (95%CI)	*p*
Stressful life event exposure									
None	44/1133	1.00 (Reference)		1.00 (Reference)		1.00 (Reference)		1.00 (Reference)	
Childhood only	53/713	1.99 (1.32, 3.00)	0.001 *	1.88 (1.24, 2.84)	0.003 *	1.85 (1.22, 2.81)	0.004 *	1.83 (1.21, 2.78)	0.004 *
Adulthood only	69/821	2.27 (1.54, 3.35)	<0.001 *	2.00 (1.34, 2.97)	<0.001 *	2.05 (1.37, 3.08)	<0.001 *	1.95 (1.30, 2.93)	0.001 *
Childhood and adulthood	75/691	3.01 (2.05, 4.43)	<0.001 *	2.52 (1.70, 3.74)	<0.001 *	2.61 (1.73, 3.93)	<0.001 *	2.52 (1.66, 3.81)	<0.001 *

OR, odds ratio; CI, confidence interval; * *p* < 0.05. Crude model: unadjusted (no covariates included). Model 1: adjusted for age and sex. Model 2: further adjusted for region, marital status, education, smoking, drinking, and body mass index (BMI). Model 3: further adjusted for hypertension, diabetes, dyslipidemia, and stroke.

## Data Availability

The data utilized in this research can be accessed publicly on the China Health and Retirement Longitudinal Study (CHARLS) website at https://charls.pku.edu.cn/.

## Supplementary Materials

The following supporting information can be downloaded at https://www.mdpi.com/article/10.3390/healthcare14203438/s1: Figure S1: Q-Q plots of age (a) and body mass index (b) for included and excluded participants; Figure S2: Q-Q plots of age (a) and body mass index (b) stratified by SLE exposure status among included participants; Figure S3: Mediation analysis for the association between SLE exposure and incident CKD through frailty (unadjusted sensitivity model); Table S1: The distribution of childhood and adulthood stressful life events in the study population; Table S2: The 32 health deficit items of the frailty index; Table S3: Comparisons of baseline characteristics between included and excluded participants; Table S4: Proportion of missing values for all study variables; Table S5: Generalized variance inflation factor (GVIF) for covariates in the logistic regression; Table S6: Generalized variance inflation factor (GVIF) for covariates in the logistic regression incorporating the frailty index; Table S7: Sensitivity analysis 1: the association between stressful life event exposure and incident chronic kidney disease at follow-up using complete-case non-imputed dataset; Table S8: Sensitivity analysis 2: using self-report of incident chronic kidney disease; Table S9: Sensitivity analysis 3: excluding participants with cancer at baseline.

## Abbreviations

SLEs	stressful life events
CKD	chronic kidney disease
CHARLS	China Health and Retirement Longitudinal Study
eGFR	estimated glomerular filtration rate
FI	frailty index
CKD-EPI	Chronic Kidney Disease Epidemiology Collaboration
BMI	body mass index
SBP	systolic blood pressure
DBP	diastolic blood pressure
FBG	fasting blood glucose
HbA1c	hemoglobin A1c
TC	total cholesterol
TG	triglycerides
LDL-C	low-density lipoprotein cholesterol
HDL-C	high-density lipoprotein cholesterol
Q-Q	quantile–quantile
IQRs	interquartile ranges
MCAR	missing completely at random
ORs	odds ratios
CIs	confidence intervals
GVIF	generalized variance inflation factors
ACME	average causal mediation effect
ADE	average direct effect
RD	risk differences
STROBE	Strengthening the Reporting of Observational Studies in Epidemiology
HPA	hypothalamic–pituitary–adrenal
RAAS	renin–angiotensin–aldosterone system
IL-6	interleukin-6
TNF-α	tumour necrosis factor-α
hs-CRP	high-sensitivity C-reactive protein
KDIGO	Kidney Disease: Improving Global Outcomes
NSAIDs	non-steroidal anti-inflammatory drugs
DAG	directed acyclic graph
RR	risk ratio
