# Supplementary material for: Association of Life-Course Stressful Life Events with Incident Chronic Kidney Disease: The Potential Mediating Role of Frailty
Source: Healthcare (Basel). 2026 Oct 14;14(20):3438. doi: 10.3390/healthcare14203438 (PMC13704301; doi:10.3390/healthcare14203438)
Supplement: Supplementary file 1 [file healthcare-14-03438-s001.zip › healthcare-4575780-supplementary.pdf]

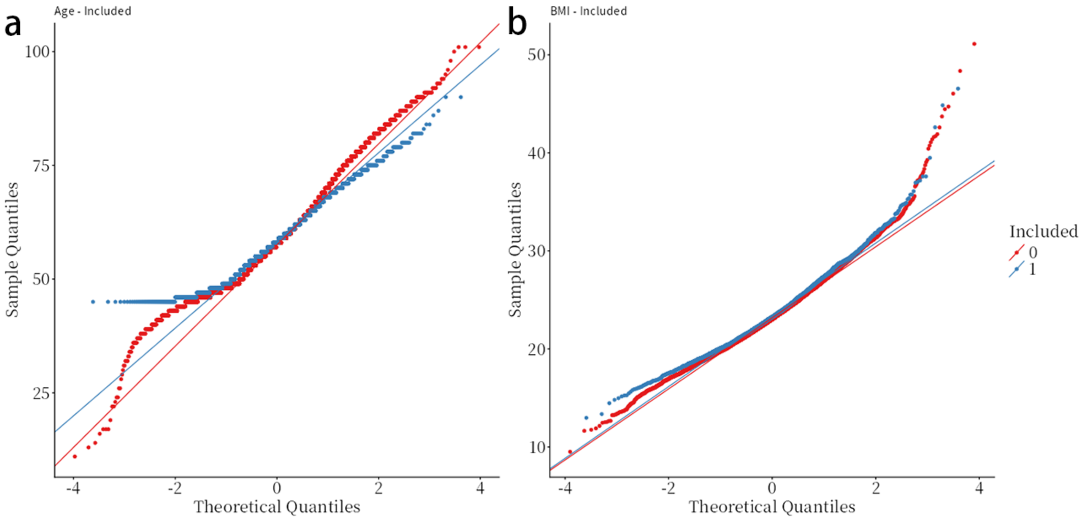

Figure S1. Q-Q plots of age (a) and body mass index (b) for included and excluded participants. Red points (0) represent participants excluded from the final analytical cohort, and blue points (1) represent participants retained in the analytical sample. BMI: body mass index.

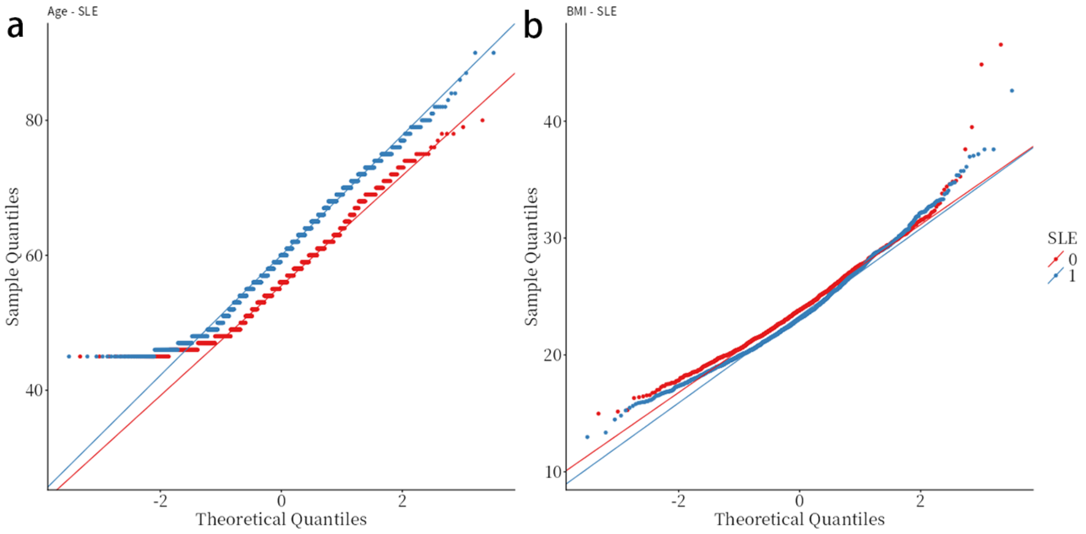

Figure S2. Q-Q plots of age (a) and body mass index (b) stratified by SLE exposure status among included participants. Red points (0) represent participants without SLE exposure; blue points (1) represent participants with SLE exposure. BMI, body mass index; SLE, stressful life event.

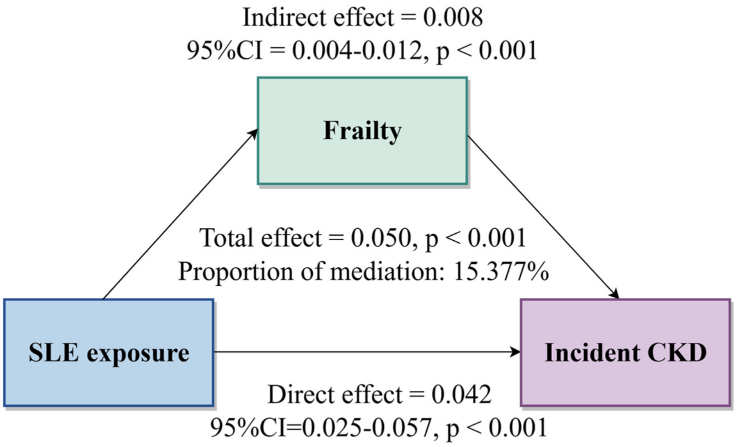

Figure S3. Mediation analysis for the association between SLE exposure and incident CKD through frailty (unadjusted sensitivity model). CKD, chronic kidney disease; SLE, stressful life event. This figure was created by Figdraw (www.figdraw.com) (ID: RYTIO062ea).

Table S1. The distribution of childhood and adulthood stressful life events in the study population.

Life stage	Stressful life event	Sample size	Yes, n (%)	No, n (%)	Missing, n (%)
Adulthood	Death of a spouse or partner	3,358	301（9.0）	3,057（91.0）	0（0.0）
	Unemployment	3,358	11（0.3）	3,347（99.7）	0（0.0）
	Life-threatening illness or accident	3,358	348（10.4）	3,010（89.6）	0（0.0）
	Death of a child	3,358	255（7.6）	3,103（92.4）	0（0.0）
Childhood	Physical attack or injury	3,358	1,052（31.3）	2,306（68.7）	0（0.0）
	Financial hardship	3,351	785（23.4）	2,566（76.6）	7（0.2）
	Parental unemployment	608	15（2.5）	593（97.5）	2,750（81.9）
	Parental substance abuse	3,333	256（7.7）	3,077（92.3）	25（0.7）
	Placement in foster care	3,357	41（1.2）	3,316（98.8）	1（0.0）
	Parental separation or divorce	3,342	29（0.9）	3,313（99.1）	16（0.5）
	Parental death	2,823	637（22.6）	2,186（77.4）	535（15.9）

Table S2. The 32 health deficit items of the frailty index.

No	Description of the items	Cut-off value
1	Self-reported physician diagnosed hypertension	Yes = 1, No = 0
2	Self-reported physician diagnosed diabetes	Yes = 1, No = 0
3	Self-reported physician diagnosed heart disease	Yes = 1, No = 0
4	Self-reported physician diagnosed stroke	Yes = 1, No = 0
5	Self-reported physician diagnosed cancer	Yes = 1, No = 0
6	Self-reported physician diagnosed arthritis	Yes = 1, No = 0
7	Self-reported physician diagnosed chronic lung disease	Yes = 1, No = 0
8	Self-reported physician diagnosed asthma	Yes = 1, No = 0
9	Self-reported physician diagnosed any emotional, nervous, or psychiatric problems	Yes = 1, No = 0
10	Self-reported physician diagnosed memory-related disease	Yes = 1, No = 0
11	Self-reported vision problems	Yes = 1, No = 0
12	Self-reported hearing problems	Yes = 1, No = 0
13	Self-reported general health status	Poor or fair = 1, excellent, very good, or good = 0
14	Difficulty with dressing	Yes = 1, No = 0
15	Difficulty with bathing or showering	Yes = 1, No = 0
16	Difficulty with eating	Yes = 1, No = 0
17	Difficulty with getting in and out of bed	Yes = 1, No = 0
18	Difficulty with using the toilet	Yes = 1, No = 0
19	Difficulty with managing money	Yes = 1, No = 0
20	Difficulty with taking medications	Yes = 1, No = 0
21	Difficulty with shopping for groceries	Yes = 1, No = 0
22	Difficulty with preparing meals	Yes = 1, No = 0

23	Difficulty with doing housework	Yes = 1, No = 0
24	Mobility: difficulty with walking 100 yards	Yes = 1, No = 0
25	Mobility: difficulty with getting up from a chair after sitting for long periods	Yes = 1, No = 0
26	Mobility: difficulty with climbing several flights of stairs without resting	Yes = 1, No = 0
27	Mobility: difficulty with lifting or carrying weights over 10 pounds/jins	Yes = 1, No = 0
28	Mobility: difficulty with picking up a coin from the table	Yes = 1, No = 0
29	Mobility: difficulty with stooping, kneeling, or crouching	Yes = 1, No = 0
30	Mobility: difficulty with reaching arms above shoulder level	Yes = 1, No = 0
31	Depression: CESD-10 questionnaire	CESD-10 $\leq$10 =0 $>$10 =1
32	Cognition: (memory test score + orientation test score) / 14	Continuous, ranging from 0 to 1

Depressive symptoms were evaluated using the Center for Epidemiologic Studies Depression Scale (CESD). The CESD-10 was adopted in China Health and Retirement Longitudinal Study (CHARLS), with total scores ranging from 0 to 30. The memory score represented the average number of unrecalled words in immediate and delayed word recall tasks, ranging from 0 to 10. The orientation test consisted of four questions regarding the day of the week, month, date, and year. One point was awarded for each incorrect answer, with scores ranging from 0 to 4. CESD, Center for Epidemiologic Studies Depression Scale.

Table S3. Comparisons of baseline characteristics between included and excluded participants.

**Variables**	**Total (n = 17,708)**	**Excluded (n = 14,350)**	**Included (n = 3,358)**	***p***
Age, year, Median (Q_1, Q_3)	58.00 (51.00, 65.00)	58.00 (50.00, 65.00)	58.00 (52.00, 65.00)	0.074
BMI, kg/m^2, Median (Q_1, Q_3)	23.09 (20.79, 25.70)	23.04 (20.73, 25.62)	23.28 (21.01, 25.95)	$<0.001^*$
Sex, n(%)				0.972
Female	9,227 (52.11)	7,478 (52.11)	1,749 (52.08)	

Male	8,479 (47.89)	6,870 (47.87)	1,609 (47.92)	
Region, n(%)				0.233
Eastern China	7,878 (44.49)	6,357 (44.30)	1,521 (45.29)	
Central China	2,761 (15.59)	2,216 (15.44)	545 (16.23)	
Western China	5,763 (32.54)	4,718 (32.88)	1,045 (31.12)	
Northeast China	1,306 (7.38)	1,059 (7.38)	247 (7.36)	
Marital status, n(%)				<0.001*
Married	15,417 (87.22)	12,403 (86.43)	3,014 (89.76)	
Other	2,258 (12.78)	1,914 (13.34)	344 (10.24)	
Education, n(%)				<0.001*
Below primary school	8,385 (47.39)	6,865 (47.84)	1,520 (45.27)	
Primary school	3,669 (20.74)	2,880 (20.07)	789 (23.50)	
Junior middle school	3,471 (19.62)	2,759 (19.23)	712 (21.20)	
Senior middle school and above	2,168 (12.25)	1,831 (12.76)	337 (10.04)	
Smoking, n(%)				0.841
No	10,628 (60.53)	8,591 (59.87)	2,037 (60.66)	
Yes	6,931 (39.47)	5,611 (39.10)	1,320 (39.31)	
Drinking, n(%)				0.361
No	10,718 (61.10)	8,690 (60.56)	2,028 (60.39)	
Yes	6,823 (38.90)	5,494 (38.29)	1,329 (39.58)	
Hypertension, n(%)				<0.001*
No	8,037 (54.30)	6,233 (43.44)	1,804 (53.72)	
Yes	6,765 (45.70)	5,426 (37.81)	1,339 (39.87)	
Diabetes, n(%)				<0.001*
No	8,534 (79.73)	5,980 (41.67)	2,554 (76.06)	
Yes	2,169 (20.27)	1,658 (11.55)	511 (15.22)	
Dyslipidemia, n(%)				<0.001*
No	6,346 (52.65)	4,497 (31.34)	1,849 (55.06)	
Yes	5,707 (47.35)	4,219 (29.40)	1,488 (44.31)	
Stroke, n(%)				<0.001*
No	16,986 (96.92)	13,699 (95.46)	3,287 (97.89)	
Yes	540 (3.08)	469 (3.27)	71 (2.11)	

Q_1, 1st Quartile; Q_3, 3rd Quartile; *, $p < 0.05$.

Table S4. Proportion of missing values for all study variables.

Variables	Total	Freq	Miss (Percent)
Age	3,358	3,358	0 (0.00)
Body mass index	3,358	3,021	337 (10.04)
Sex	3,358	3,358	0 (0.00)
Region	3,358	3,358	0 (0.00)
Marital status	3,358	3,358	0 (0.00)
Education	3,358	3,358	0 (0.00)
Smoking	3,358	3,357	1 (0.03)
Drinking	3,358	3,357	1 (0.03)
Hypertension	3,358	3,143	215(6.40)
Diabetes	3,358	3,065	293(8.73)
Dyslipidemia	3,358	3,337	21(0.63)
Stroke	3,358	3,358	0 (0.00)

Freq, number of non-missing observations; Miss (Percent), number (percentage) of missing values.

Table S5. Generalized variance inflation factor (GVIF) for covariates in the logistic regression.

Variables	GVIF	Df	$GVIF^{1/(2*Df)}$
Stressful life event exposure	1.124	1	1.060
Age	1.293	1	1.137
BMI	1.272	1	1.128
Sex	2.268	1	1.506
Region	1.061	3	1.010
Marital status	1.131	1	1.063
Education	1.270	3	1.041
Smoking	1.915	1	1.384
Drinking	1.446	1	1.203

Variables	GVIF	Df	$GVIF^{1/(2*Df)}$
Hypertension	1.158	1	1.076
Diabetes	1.063	1	1.031
Dyslipidemia	1.124	1	1.060
Stroke	1.021	1	1.010

BMI, body mass index; Df, degrees of freedom; GVIF, generalized variance inflation factor.

Table S6. Generalized variance inflation factor (GVIF) for covariates in the logistic regression incorporating the frailty index.

Variables	GVIF	Df	$GVIF^{1/(2*Df)}$
Stressful life event exposure	1.210	1	1.100
Age	1.305	1	1.143
BMI	1.272	1	1.128
Sex	2.327	1	1.525
Region	1.073	3	1.012
Marital status	1.130	1	1.063
Education	1.269	3	1.040
Smoking	1.916	1	1.384
Drinking	1.465	1	1.210
Hypertension	1.194	1	1.093
Diabetes	1.072	1	1.035
Dyslipidemia	1.120	1	1.059
Stroke	1.042	1	1.021
Frailty index	1.279	1	1.131

BMI, body mass index; Df, degrees of freedom; GVIF, generalized variance inflation factor.

Table S7. Sensitivity analysis 1: The association between stressful life event exposure and incident chronic kidney disease at follow-up using complete-case non-imputed dataset.

Variables	Crude model	Model 1	Model 2	Model 3

	OR (95%CI)	p	OR (95%CI)	p	OR (95%CI)	p	OR (95%CI)	p
**Stressful life event exposure**								
No	1.00 (Reference)		1.00 (Reference)		1.00 (Reference)		1.00 (Reference)	
Yes	2.40 (1.72, 3.36)	<0.001*	1.86 (1.30, 2.65)	<0.001*	1.90 (1.29, 2.79)	0.001*	1.85 (1.26, 2.72)	0.002*
**Count of stressful life events**								
Per stressful life event	1.37 (1.22, 1.53)	<0.001*	1.26 (1.11, 1.43)	<0.001*	1.33 (1.16, 1.54)	<0.001*	1.33 (1.15, 1.54)	<0.001*
0	1.00 (Reference)		1.00 (Reference)		1.00 (Reference)		1.00 (Reference)	
1	2.00 (1.38, 2.90)	<0.001*	1.53 (1.03, 2.28)	0.035*	1.52 (1.00, 2.32)	0.052	1.48 (0.97, 2.26)	0.071
≥2	2.88 (2.00, 4.13)	<0.001*	2.25 (1.53, 3.32)	<0.001*	2.50 (1.64, 3.81)	<0.001*	2.44 (1.60, 3.73)	<0.001*
p for trend	<0.001*		<0.001*		<0.001*		<0.001*	
**Childhood stressful life event exposure**								
No	1.00 (Reference)		1.00 (Reference)		1.00 (Reference)		1.00 (Reference)	
Yes	1.63 (1.26, 2.13)	<0.001*	1.52 (1.14, 2.02)	0.004*	1.51 (1.11, 2.04)	0.008*	1.52 (1.12, 2.06)	0.007*
**Count of childhood stressful life events**								
Per stressful life event	1.40 (1.18, 1.67)	<0.001*	1.35 (1.12, 1.63)	0.002*	1.35 (1.10, 1.66)	0.004*	1.36 (1.11, 1.66)	0.003*
0	1.00 (Reference)		1.00 (Reference)		1.00 (Reference)		1.00 (Reference)	
1	1.52 (1.15, 2.02)	0.004*	1.41 (1.03, 1.91)	0.030*	1.39 (1.01, 1.93)	0.048*	1.41 (1.01, 1.95)	0.042*
≥2	2.04 (1.36, 3.06)	<0.001*	1.93 (1.26, 2.98)	0.003*	1.94 (1.23, 3.06)	0.004*	1.94 (1.23, 3.06)	0.005*
p for trend	<0.001*		0.001*		0.002*		0.002*	
**Adulthood stressful life event exposure**								
No	1.00 (Reference)		1.00 (Reference)		1.00 (Reference)		1.00 (Reference)	

Variables	Crude model		Model 1		Model 2		Model 3	
	OR (95%CI)	p	OR (95%CI)	p	OR (95%CI)	p	OR (95%CI)	p
Yes	1.90 (1.45, 2.48)	<0.001*	1.57 (1.17, 2.11)	0.003*	1.71 (1.24, 2.36)	0.001*	1.66 (1.20, 2.29)	0.002*
**Count of adulthood stressful life events**								
Per stressful life event	1.42 (1.21, 1.67)	<0.001*	1.23 (1.03, 1.48)	0.025*	1.35 (1.09, 1.66)	0.005*	1.33 (1.08, 1.65)	0.008*
0	1.00 (Reference)		1.00 (Reference)		1.00 (Reference)		1.00 (Reference)	
1	1.81 (1.36, 2.42)	<0.001*	1.56 (1.14, 2.14)	0.005*	1.66 (1.19, 2.32)	0.003*	1.61 (1.15, 2.25)	0.006*
≥2	2.15 (1.47, 3.15)	<0.001*	1.60 (1.04, 2.46)	0.033*	1.93 (1.19, 3.14)	0.008*	1.89 (1.17, 3.08)	0.010*
p for trend	<0.001*		0.007*		0.001*		0.002*	

OR, Odds Ratio; CI, Confidence Interval; *, $p < 0.05$.
Crude model: unadjusted (no covariates included).
Model 1: adjusted for age and sex.
Model 2: further adjusted for region, marital status, education, smoking, drinking, and body mass index (BMI).
Model 3: further adjusted for hypertension, diabetes, dyslipidemia, and stroke.

Table S8. Sensitivity analysis 2: Using self-report of incident chronic kidney disease.

Variables	Events/total, n	Crude model		Model 1		Model 2		Model 3	
		OR (95%CI)	p	OR (95%CI)	p	OR (95%CI)	p	OR (95%CI)	p
**Stressful life event exposure**									
No	29/1,133	1.00 (Reference)		1.00 (Reference)		1.00 (Reference)		1.00 (Reference)	
Yes	140/2,225	2.56 (1.70, 3.84)	<0.001*	2.56 (1.69, 3.86)	<0.001*	2.48 (1.63, 3.78)	<0.001*	2.42 (1.59, 3.70)	<0.001*
**Count of stressful life events**									
Per stressful life event		1.34 (1.17, 1.53)	<0.001*	1.34 (1.17, 1.54)	<0.001*	1.34 (1.15, 1.55)	<0.001*	1.33 (1.15, 1.55)	<0.001*
0	29/1,133	1.00 (Reference)		1.00 (Reference)		1.00 (Reference)		1.00 (Reference)	

Variables	Events/total, n	Crude model		Model 1		Model 2		Model 3	
		OR (95%CI)	*p*	OR (95%CI)	*p*	OR (95%CI)	*p*	OR (95%CI)	*p*
1	67/1,188	2.28 (1.46, 3.55)	<0.001*	2.29 (1.47, 3.58)	<0.001*	2.24 (1.42, 3.51)	<0.001*	2.19 (1.39, 3.44)	<0.001*
≥2	73/1,037	2.88 (1.86, 4.47)	<0.001*	2.91 (1.86, 4.55)	<0.001*	2.87 (1.80, 4.58)	<0.001*	2.80 (1.75, 4.48)	<0.001*
p for trend		<0.001*		<0.001*		<0.001*		<0.001*	
**Childhood stressful life event exposure**									
No	78/1,954	1.00 (Reference)		1.00 (Reference)		1.00 (Reference)		1.00 (Reference)	
Yes	91/1,404	1.67 (1.22, 2.27)	0.001*	1.63 (1.19, 2.23)	0.002*	1.55 (1.13, 2.13)	0.007*	1.55 (1.13, 2.14)	0.007*
**Count of childhood stressful life events**									
Per stressful life event		1.42 (1.15, 1.74)	<0.001*	1.39 (1.13, 1.71)	0.002*	1.34 (1.08, 1.66)	0.007*	1.35 (1.09, 1.67)	0.007*
0	78/1,954	1.00 (Reference)		1.00 (Reference)		1.00 (Reference)		1.00 (Reference)	
1	67/1,099	1.56 (1.12, 2.18)	0.009*	1.54 (1.10, 2.15)	0.013*	1.47 (1.05, 2.07)	0.026*	1.48 (1.05, 2.08)	0.026*
≥2	24/305	2.05 (1.28, 3.30)	0.003*	1.96 (1.22, 3.16)	0.006*	1.84 (1.13, 2.99)	0.015*	1.84 (1.13, 3.00)	0.015*
p for trend		<0.001*		0.001*		0.005*		0.005*	
**Adulthood stressful life event exposure**									
No	72/1,846	1.00 (Reference)		1.00 (Reference)		1.00 (Reference)		1.00 (Reference)	
Yes	97/1,512	1.69 (1.24, 2.31)	0.001*	1.70 (1.23, 2.35)	0.001*	1.67 (1.20, 2.35)	0.003*	1.63 (1.16, 2.28)	0.005*
**Count of adulthood stressful life events**									
Per stressful life event		1.34 (1.11, 1.62)	0.002*	1.36 (1.11, 1.66)	0.003*	1.37 (1.10, 1.70)	0.005*	1.35 (1.08, 1.68)	0.008*
0	72/1,846	1.00 (Reference)		1.00 (Reference)		1.00 (Reference)		1.00 (Reference)	
1	68/1,118	1.60 (1.14, 2.24)	0.007*	1.61 (1.14, 2.27)	0.007*	1.59 (1.11, 2.26)	0.010*	1.54 (1.08, 2.20)	0.018*

Variables	Events/total, n	Crude model		Model 1		Model 2		Model 3	
		OR (95%CI)	p	OR (95%CI)	p	OR (95%CI)	p	OR (95%CI)	p
≥2	29/394	1.96 (1.25, 3.06)	0.003*	2.01 (1.27, 3.20)	0.003*	2.05 (1.25, 3.38)	0.005*	2.00 (1.21, 3.30)	0.007*
p for trend		<0.001*		<0.001*		0.001*		0.003*	

OR, Odds Ratio; CI, Confidence Interval; *, $p < 0.05$.
Crude model: unadjusted (no covariates included).
Model 1: adjusted for age and sex.
Model 2: further adjusted for region, marital status, education, smoking, drinking, and body mass index (BMI).
Model 3: further adjusted for hypertension, diabetes, dyslipidemia, and stroke.

Table S9. Sensitivity analysis 3: Excluding participants with cancer at baseline.

Variables	Events/total, n	Crude model		Model 1		Model 2		Model 3	
		OR (95%CI)	p	OR (95%CI)	p	OR (95%CI)	p	OR (95%CI)	p
**Stressful life event exposure**									
No	42/1,121	1.00 (Reference)		1.00 (Reference)		1.00 (Reference)		1.00 (Reference)	
Yes	196/2,208	2.50 (1.78, 3.52)	<0.001*	2.19 (1.55, 3.10)	<0.001*	2.26 (1.59, 3.22)	<0.001*	2.20 (1.54, 3.13)	<0.001*
**Count of stressful life events**									
Per stressful life event		1.38 (1.24, 1.55)	<0.001*	1.29 (1.15, 1.45)	<0.001*	1.31 (1.16, 1.49)	<0.001*	1.31 (1.15, 1.49)	<0.001*
0	42/1,121	1.00 (Reference)		1.00 (Reference)		1.00 (Reference)		1.00 (Reference)	
1	88/1,179	2.07 (1.42, 3.02)	<0.001*	1.91 (1.30, 2.79)	<0.001*	1.89 (1.29, 2.78)	0.001*	1.84 (1.25, 2.70)	0.002*
≥2	108/1,029	3.01 (2.09, 4.35)	<0.001*	2.55 (1.75, 3.72)	<0.001*	2.63 (1.78, 3.90)	<0.001*	2.55 (1.72, 3.79)	<0.001*
p for trend		<0.001*		<0.001*		<0.001*		<0.001*	
**Childhood stressful life event exposure**									
No	110/1,935	1.00 (Reference)		1.00 (Reference)		1.00 (Reference)		1.00 (Reference)	
Yes	128/1,394	1.68 (1.29, 2.19)	<0.001*	1.56 (1.19, 2.03)	0.001*	1.51 (1.15, 1.99)	0.003*	1.52 (1.15, 1.99)	0.003*

Variables	Events/total, n	Crude model		Model 1		Model 2		Model 3	
		OR (95%CI)	p	OR (95%CI)	p	OR (95%CI)	p	OR (95%CI)	p
**Count of childhood stressful life events**									
Per stressful life event		1.42 (1.19, 1.70)	<0.001*	1.35 (1.13, 1.62)	<0.001*	1.33 (1.11, 1.60)	0.002*	1.33 (1.11, 1.60)	0.002*
0	110/1,935	1.00 (Reference)		1.00 (Reference)		1.00 (Reference)		1.00 (Reference)	
1	94/1,092	1.56 (1.17, 2.08)	0.002*	1.46 (1.09, 1.95)	0.010*	1.43 (1.07, 1.91)	0.017*	1.43 (1.07, 1.92)	0.016*
≥2	34/302	2.10 (1.40, 3.16)	<0.001*	1.91 (1.27, 2.87)	0.002*	1.84 (1.21, 2.79)	0.004*	1.83 (1.21, 2.79)	0.005*
p for trend		<0.001*		<0.001*		0.001*		0.001*	
**Adulthood stressful life event exposure**									
No	95/1,831	1.00 (Reference)		1.00 (Reference)		1.00 (Reference)		1.00 (Reference)	
Yes	143/1,498	1.93 (1.47, 2.52)	<0.001*	1.68 (1.27, 2.22)	<0.001*	1.71 (1.28, 2.29)	<0.001*	1.65 (1.23, 2.21)	<0.001*
**Count of adulthood stressful life events**									
Per stressful life event		1.43 (1.22, 1.68)	<0.001*	1.28 (1.08, 1.52)	0.004*	1.33 (1.11, 1.61)	0.003*	1.31 (1.08, 1.58)	0.005*
0	95/1,831	1.00 (Reference)		1.00 (Reference)		1.00 (Reference)		1.00 (Reference)	
1	101/1,105	1.84 (1.37, 2.46)	<0.001*	1.66 (1.23, 2.23)	<0.001*	1.67 (1.24, 2.26)	<0.001*	1.61 (1.18, 2.18)	0.002*
≥2	42/393	2.19 (1.49, 3.20)	<0.001*	1.74 (1.17, 2.60)	0.007*	1.88 (1.22, 2.89)	0.004*	1.82 (1.18, 2.81)	0.007*
p for trend		<0.001*		<0.001*		<0.001*		0.001*	

OR, Odds Ratio; CI, Confidence Interval; *, $p < 0.05$.
Crude model: unadjusted (no covariates included).
Model 1: adjusted for age and sex.
Model 2: further adjusted for region, marital status, education, smoking, drinking, and body mass index (BMI).
Model 3: further adjusted for hypertension, diabetes, dyslipidemia, and stroke.

STROBE Statement—Checklist of items that should be included in reports of ***cohort studies***

	Item No	Recommendation	Y/N	Page
**Title and abstract**	1	(*a*) Indicate the study's design with a commonly used term in the title or the abstract	Y	1
		(*b*) Provide in the abstract an informative and balanced summary of what was done and what was found	Y	1
**Introduction**				
Background/rationale	2	Explain the scientific background and rationale for the investigation being reported	Y	2-3
Objectives	3	State specific objectives, including any prespecified hypotheses	Y	3
**Methods**				
Study design	4	Present key elements of study design early in the paper	Y	3
Setting	5	Describe the setting, locations, and relevant dates, including periods of recruitment, exposure, follow-up, and data collection	Y	3
Participants	6	(*a*) Give the eligibility criteria, and the sources and methods of selection of participants. Describe methods of follow-up	Y	3
		(*b*) For matched studies, give matching criteria and number of exposed and unexposed	N/A	N/A
Variables	7	Clearly define all outcomes, exposures, predictors, potential confounders, and effect modifiers. Give diagnostic criteria, if applicable	Y	4-5
Data sources/ measurement	8*	For each variable of interest, give sources of data and details of methods of assessment (measurement). Describe comparability of assessment methods if there is more than one group	Y	3
Bias	9	Describe any efforts to address potential sources of bias	Y	5-6
Study size	10	Explain how the study size was arrived at	Y	3-4
Quantitative variables	11	Explain how quantitative variables were handled in the analyses. If applicable, describe which groupings were chosen and why	Y	5
Statistical methods	12	(*a*) Describe all statistical methods, including those used to control for confounding	Y	5-6
		(*b*) Describe any methods used to examine subgroups and interactions	Y	6
		(*c*) Explain how missing data were addressed	Y	5
		(*d*) If applicable, explain how loss to follow-up was addressed	Y	3-4
		(*e*) Describe any sensitivity analyses	Y	6
**Results**				
Participants	13*	(a) Report numbers of individuals at each stage of study—eg numbers potentially eligible, examined for eligibility, confirmed eligible, included in the study, completing follow-up, and analysed	Y	3
		(b) Give reasons for non-participation at each stage	Y	3
		(c) Consider use of a flow diagram	Y	3

Descriptive data	14*	(a) Give characteristics of study participants (eg demographic, clinical, social) and information on exposures and potential confounders	Y	6-8
		(b) Indicate number of participants with missing data for each variable of interest	Y	5
		(c) Summarise follow-up time (eg, average and total amount)	Y	3
Outcome data	15*	Report numbers of outcome events or summary measures over time	Y	9
Main results	16	(*a*) Give unadjusted estimates and, if applicable, confounder-adjusted estimates and their precision (eg, 95% confidence interval). Make clear which confounders were adjusted for and why they were included	Y	9-11
		(*b*) Report category boundaries when continuous variables were categorized	Y	4
		(*c*) If relevant, consider translating estimates of relative risk into absolute risk for a meaningful time period	N/A	N/A
Other analyses	17	Report other analyses done—eg analyses of subgroups and interactions, and sensitivity analyses	Y	11-12
**Discussion**				
Key results	18	Summarise key results with reference to study objectives	Y	12
Limitations	19	Discuss limitations of the study, taking into account sources of potential bias or imprecision. Discuss both direction and magnitude of any potential bias	Y	15-16
Interpretation	20	Give a cautious overall interpretation of results considering objectives, limitations, multiplicity of analyses, results from similar studies, and other relevant evidence	Y	17
Generalisability	21	Discuss the generalisability (external validity) of the study results	Y	14-16
**Other information**				
Funding	22	Give the source of funding and the role of the funders for the present study and, if applicable, for the original study on which the present article is based	Y	17

*Give information separately for exposed and unexposed groups.

Note: An Explanation and Elaboration article discusses each checklist item and gives methodological background and published examples of transparent reporting. The STROBE checklist is best used in conjunction with this article (freely available on the Web sites of PLoS Medicine at http://www.plosmedicine.org/, Annals of Internal Medicine at http://www.annals.org/, and Epidemiology at http://www.epidem.com/). Information on the STROBE Initiative is available at http://www.strobe-statement.org.
